# Phase-controlled field-effect micromixing using AC electroosmosis

**DOI:** 10.1038/s41378-020-0166-y

**Published:** 2020-07-27

**Authors:** Paresa Modarres, Maryam Tabrizian

**Affiliations:** 1 Biomedical Engineering Department, McGill University, Montreal, QC Canada; 2 Faculty of Dentistry, McGill University, 2001 McGill College Ave, Montreal, QC Canada

**Keywords:** Nanoparticles, Microfluidics

## Abstract

The exploration and application of electrokinetic techniques in micro total analysis systems have become ubiquitous in recent years, and scientists are expanding the use of such techniques in areas where comparable active or passive methods are not as successful. In this work, for the first time, we utilize the concept of AC electroosmosis to design a phase-controlled field-effect micromixer that benefits from a three-finger sinusoidally shaped electrodes. Analogous to field-effect transistor devices, the principle of operation for the proposed micromixer is governed by the source-gate and source-drain voltage potentials that are modulated by introducing a phase lag between the driving electrodes. At an optimized flow rate and biasing scheme, we demonstrate that the source, gate, and drain voltage phase relations can be configured such that the micromixer switches from an unmixed state (phase shift of 0°) to a mixed state (phase shift of 180°). High mixing efficiencies beyond 90% was achieved at a volumetric flow rate of 4 µL/min corresponding to ~13.9 mm/s at optimized voltage excitation conditions. Finally, we employed the proposed micromixer for the synthesis of nanoscale lipid-based drug delivery vesicles through the process of electrohydrodynamic-mediated nanoprecipitation. The phase-controlled electrohydrodynamic mixing utilized for the nanoprecipitation technique proved that nanoparticles of improved monodispersity and concentration can be produced when mixing efficiency is enhanced by tuning the phase shifts between electrodes.

## Microfluidics: A novel micromixer based on AC electroosmosis

Researchers in Canada have developed a device which can efficiently mix water in microfluidic devices. The dominance of viscosity in the microscale dynamics means that mixing only occurs via diffusion, making micromixing devices a key tool for working at these scales. Paresa Modarres and Maryam Tabrizian at McGill University engineered an active mixing device consisting of three sinusoidal electrodes running through the water channel. Applying a voltage across these electrodes generates an electric field which causes the water streams to mix. By varying the phase of the electrodes, they could control the strength of mixing, switching the micromixer between a mixing and non-mixing state. Finally, the duo tests the new device by using it to synthesize lipid nano-vesicles from a mixed solution of water and lipid-containing ethanol.

## Introduction

For the past two decades, one of the foremost applications of microfabrication technology in biomedical sciences has been the advent of microfluidic devices for handling minute amounts of biological liquids with broad applicability to analytical and diagnostic devices. While miniaturization is beneficial in many aspects such as low volume consumption, portability, and accessibility, it poses new challenges due to fluid physics at the microscale. At microscale dimensions, the dominance of viscous to inertial forces leads to laminar flows wherein mixing is only possible by diffusion. However, with diffusion coefficients ranging from 10^−9^ to 10^−11^ m^2^/s depending on the molecule size, mixing by diffusion is extremely slow and inefficient. Consequently, many passive and active micromixers have emerged to enhance mixing within microfluidic channels. Passive micromixers rely on the fluid transport mechanism to promote mixing by stretching the interface between the liquids and reducing the striation length across which diffusion takes place. Examples of passive micromixers include hydrodynamic focusers^1^, lamination-based designs^2,3^, and those implementing geometrical obstacles^4–7^. Passive micromixers are simple to operate but the fabrication process can become very complex especially for the three-dimensional designs^8^. Furthermore, many passive techniques suffer from sample dispersion and dilution by spreading it out longitudinally compromising assay sensitivity and microenvironment homogenity^9^. Active micromixers do not suffer from the dilution problem since these mixers maintain a constant volume during the mixing process by the use of an external force source such as magnetic^10–14^, acoustic^15–17^, and electrical^18–25^ to drive local fluid flow. Amongst active micromixers, electrical-based methods offer unique properties such as the ease of electrode implementation, the absence of any moving parts, and the use of small voltages making them an attractive mixing mechanism for many bioanalytical applications. Most significantly, electrical-based mixing is an excellent choice for mixing in droplet-based microfluidics^19,26^ and sensitivity enhancement by overcoming diffusion-limited analyte transport in several sensing platforms^27^ like impedance sensing^28–30^, plasmonic sensing^31^, and quartz crystal microbalance^32^ by fabricating additional electrodes or modifying the sensing electrodes.

Microfluidic-based electrokinetic mixers typically embed electrodes within microfluidic channels that cause fluid motion upon voltage excitation. The mechanism of fluid motion and the strength of induced flows are highly dependent on the electrical parameters of the fluid (i.e., conductivity and permittivity), monophasic or multiphasic nature of the liquid system, and voltage parameters (i.e., AC frequency and voltage amplitude). Application of low-frequency AC voltages to a pair of co-planar electrodes in contact with an electrolyte generates steady (non-zero time-averaged) fluid motion that is driven by the interaction of electric field with its self-induced charges in the electrical double layer (EDL)^33^. This type of flow is referred to as AC electroosmosis (ACEO) and is a strong function of the tangential electric field and the zeta potential^34^. In this context, zeta potential is defined as the voltage at the edge of the shearing plane on the surface of electrodes to the bulk medium. The ACEO fluid velocity is strongest for fluids with low ionic conductivities, and peaks at frequencies below the reciprocal charge relaxation time of the fluid (typically in the range of hundreds of Hz to tens of kHz)^35^ {Green, 2000 #497}. On the contrary, fluids with high conductivities can undergo Joule heating when AC voltages of high frequency (>100 kHz) with large amplitudes are utilized. The temperature gradients as a result of Joule heating cause local conductivity and permittivity gradients creating volume space charges which are the source of AC electrothermal (ACET) fluid actuation in the presence of a non-uniform electric field^36^. The ACEO and ACET flows are well characterized for liquid systems of homogenous compositions. When electric fields are applied across media consisting of multiple liquids of distinct electrical permittivity/conductivity parameters, the Maxwell stresses acting on the accumulated monopolar charges at the interface derives fluid motion^37,38^. This type of flow is referred to as electrohydrodynamics, which has developed separately from electrokinetic phenomena although both electrokinetics and electrohydrodynamics are concerned with electric-field induced fluid motion.

The integration of AC electrokinetic techniques within microfluidic channels initiated with the pioneering studies by Green and colleagues that experimentally and theoretically investigated fluid actuation by ACEO^33,35,39^. Later on, ACEO induced flows were implemented in applications involving pumping^40^, mixing^41^, and analyte transport^42^ for various bioanalytical systems. Within the domain of micromixing, coplanar electrodes generating transverse electric fields with respect to the incoming flow direction were utilized for efficient mixing of low conductivity fluid streams by ACEO^22–24,43,44^. Those micromixers mostly employed coplanar electrode pairs, which are limited in providing flow control capability and field enhancement through various biasing configurations. If an additional electrode at fixed potential is added, the magnitude and direction of flow can further be controlled by introducing potential or phase imbalances between electrodes to realize a rich variety of effects that do not occur when only one electrode pair is used. Consequently, the AC signals in a three-electrode micromixer can be tunned such that the micromixer operates analogously to the field-effect transistors (FETs) whereby the gate, source, and drain voltages are set to have a conducting/non-conducting channel in linear mode. Likewise, in an electrokinetic micromixer with electrodes in contact with a solution, the voltage on each electrode defines the electric field intensity and distribution that enable different levels of fluid mixing.

The field-effect concept for fluid handling has mostly been applied in conventional electroosmosis for directional flow control and pumping by the application of a perpendicular electric field to the channel surface enabling modification of zeta potential^45–48^. However, field-effect electroosmotic flows are very sensitive to the pH of the solution and are ineffective at pH values higher or lower than the value at which the native zeta potential of the surface is zero. Moreover, they mostly operate by the application of large DC voltages across the channel that are prohibitively prone to electrolysis and bubble formation. Recently, Wu et al. demonstrated a FET-based AC electroosmotic micromixer using two sets of three-electrodes in a tandem configuration^18^. They demonstrated that by tuning the gate electrodes voltages, asymmetric vortices are generated, and mixing degree can be adjusted by a small degree. However, the use of six electrodes unnecessarily complicates device biasing and analysis. Furthermore, the mixing performance in a voltage-tuned micromixer is compromised when the maximum allowed voltage is limited.

**Fig. 1:**
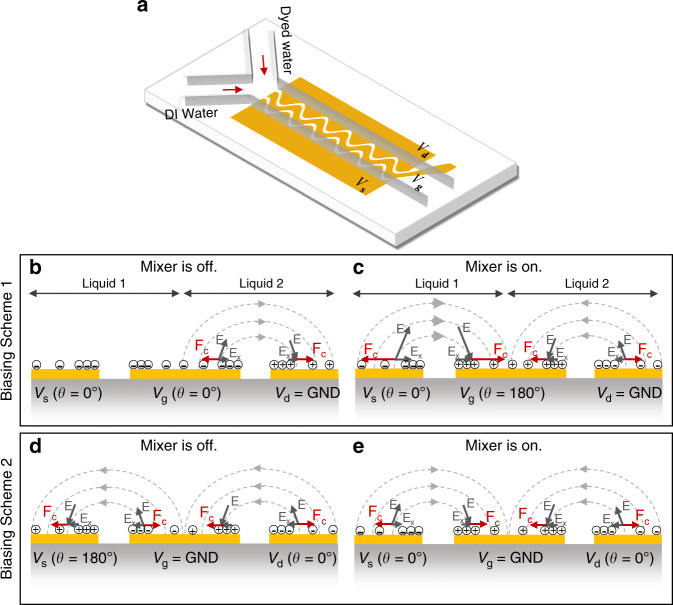
Schematic illustration of device structure and working principle. **a** Schematic illustration of the micromixer. **b** Minimized and **c** maximized mixing occur in biasing scheme 1 when Δ*θ*_Vgs_ = 0° and Δ*θ*_Vgs_ = 180°, respectively. For the biasing scheme 2, **d** minimized and **e** maximized mixing take place when Δ*θ*_Vds_ = 180° and Δ*θ*_Vds_ = 0°, respectively

Herein, we provide the first demonstration of a phase-controlled electrically powered micromixer whereby the phase shift between electrodes regulates the mixing extent from an unmixed state to a mixed state similar to that of a FET in switching mode (Fig. 1a). Applying voltages of the same amplitude with a phase lag offers flow control capability without compensating the electric potential intensities by unbalancing voltage amplitudes. We define different biasing conditions for the proposed micromixer and lay out the governing rules for achieving the best mixing by considering the source-gate and the source-drain voltages. The insightful operational analogy between the field-effect micromixer and a FET device, delivered in this work, greatly simplifies the analysis of the proposed micromixer. Finally, we utilize the proposed micromixer for the generation of nanoscale lipid-based vesicles (liposomes) employing the process of nanoprecipitation (or solvent displacement). We show that rapid mixing of reagents, when optimized AC signals are applied, yields highly monodisperse nanoparticle populations.

## Theoretical background

The ACEO liquid motion is driven by the interaction of a nonuniform electric field with the induced charges in the diffuse double layer. A double layer forms on electrodes by the electric potential that moves counter-ions by electro-migration from the bulk electrolyte to the surface of electrodes. As the double layer charges, the tangential electric field component produces flow within the thin double layer that is the source of long-range flow patterns via the viscous forces. In an AC field, the flow velocity and direction remain unchanged in each half-cycle as both the electrode and the double layer reverse polarity, simultaneously. Thus, a time-average Maxwell force is generated giving rise to a steady electroosmotic slip velocity, which typically points from the inner electrode spacing over the surface of electrodes when a coplanar electrode pair is considered. In a liquid of permittivity, *ε*, conductivity, *σ*, and viscosity, *μ*, the time-averaged electroosmotic velocity at distance *x* from inner electrode spacing is given as follows^34,35,49^:

1 $$v = \frac{1}{2}{\mathrm{Re}}\left\{ {\frac{{\Delta \sigma _qE_{x}^{\ast} }}{{\mu \kappa}}} \right\} = \frac{1}{8}\frac{\varepsilon V^{2}{\mathit{\Omega}}^{2}}{{{\mu} x(1 + {\mathit{\Omega}}^{2})^{2}}}\frac{{C_{\rm{s}}}}{{C_{\rm{s}} + C_{\rm{d}}}}$$

2 $${\mathit{\Omega}} = {\omega} \frac{\varepsilon }{\sigma }\frac{\pi }{2}x\kappa$$

where *V* is the amplitude of AC potential, *κ*^−*1*^ is the Debye length of the double layer, *ω* is the angular frequency, and *Ω* is the non-dimensional characteristic frequency. *E*_*x*_ is the tangential electric field component, and *Δσ* is the time-dependent excess charge in the diffuse layer. The last right-hand-side term in Eq. 1 is a correction factor with *C*_s_ being the Stern layer (or compact layer) capacitance and *C*_d_ being the diffuse layer capacitance. At high frequencies, the applied potential is entirely dropped across the media, while at low frequencies the voltage drop is across the EDL. Thus, at high frequencies, *Δ*_*σ*_ tends to zero resulting in no ACEO flow. At low frequencies, the electric field in the bulk media tends to zero; however, since the tangential electric field must be continuous, the *E*_*x*_ approaches zero in the double layer, which stops the ACEO flow. Accordingly, the ACEO flow peaks at an intermediate characteristic frequency, and ceases at high- and low-frequency limits.

## Principal of operation

The proposed micromixer consists of a Y-shaped microchannel with three-finger gold electrodes that are shaped sinusoidally (s-shape) running parallel to the main channel (Fig. 1a). The center electrode is denoted as the gate electrode, and the side electrodes are interchangeably designated as the source and drain electrodes. Two coflowing streams of deionized water (DI) and dyed water solution are introduced to the mixing channel. Upon voltage excitation, the ACEO-generated transverse flows with an asymmetric pattern along the mixing length lead to the mixing of the streams.

To understand the phase-actuating mechanism, Fig. 1b–e schematics illustrate the physical system at a given time in the first half-cycle of AC signals looking at a 2D cross-section of the device. The voltages assigned to the source, gate, and drain electrodes are designated as *V*_s_, *V*_g_, and *V*_d_, respectively. With one electrode grounded, two biasing schemes can be assumed for the mixer. In the first biasing scheme (Fig. 1b, c), the drain electrode is grounded, and the source and gate electrodes are biased with two AC signals of the same amplitude with the gate electrode imposing a phase difference. In the second biasing scheme (Fig. 1d, e), the gate electrode is grounded, and the source and drain electrodes are given two AC signals of the same amplitude with the source electrode introducing the phase shift. In both biasing conditions, the driving electrodes are given voltages of the same frequency, and only the phase difference is modulated.

**Fig. 2:**
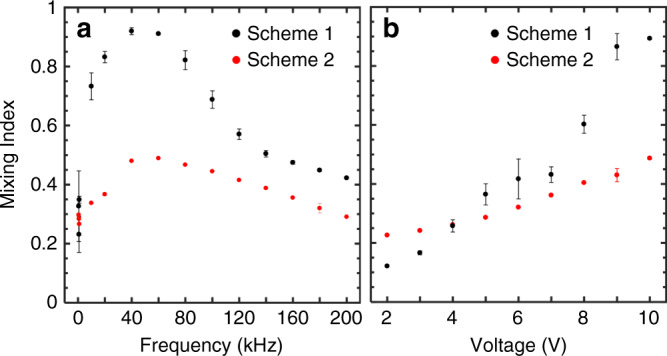
Mixing dependence on frequency and voltage. MI versus **a** frequency and **b** voltage for the two biasing schemes. All experiments were performed with a 10 V_pp_ at the optimal phase lag and a confluent flow rate of 4 µL/min

Mixing takes place at the interface of the two liquid streams by the microvortices generated at the electrode gaps that are extended over the electrode surfaces. The intensity of those microvortices dictates the degree of mixing. The micromixer in biasing scheme 1 acts like a FET in switching mode or linear regime with no mixing when the phase difference is zero (Δ*θ*_*V*gs_ = 0°) and maximized mixing when the signals are antiphase (Δ*θ*_*V*gs_ = 180°). In biasing scheme 2, a similar but opposite response to phase modulation is observed with less intensity. The difference between the two biasing schemes and the overall mixing mechanism can be explained by considering the potential difference between the electrodes and the electric field intensity distribution when the phase lag is modulated. For instance, when Δ*θ*_*V*gs_ = 0° for the first biasing condition (Fig. 1b), the potential difference between the source and gate electrodes is zero at all times leading to minimal mixing due to the lack of flow at the source-gate electrode gap. Conversely, at Δ*θ*_*V*gs_ = 180° (Fig. 2c), the potential difference between the source and gate electrodes is maximized providing optimal mixing for the given potentials. Table 1 shows the device operation under different biasing schemes considering the peak voltage in the first half-cycle of AC signals.

**Table 1:** Phase-controlled field-effect mixing under different biasing schemes

	*V*_s_	*V*_g_	*V*_d_	|*V*_gs_|	|*V*_ds_|	Phase difference	Biasing condition	State
Fig. 2b	5	5	0	0	5	$\Delta \theta _{V_{\text{gs}}} = {\mathrm{0}}^\circ$	|V_gs_| < |V_ds_|	Off
Fig. 2c	5	−5	0	10	5	$\Delta \theta _{V_{\text{gs}}} = 180^\circ$	|V_gs_| > |V_ds_|	On
Fig. 2d	−5	0	5	5	10	$\Delta \theta _{V_{\text{ds}}} = 180^\circ$	|V_gs_| < |V_ds_|	Off
Fig. 2e	5	0	5	5	0	$\Delta \theta _{V_{\text{ds}}} = {\mathrm{0}}^\circ$	|V_gs_| > |V_ds_|	On

At any flow rate and regardless of the biasing scheme, the best mixing takes place when the potential difference between the gate and source electrodes (|*V*_gs_|) is larger than the potential difference between the source and drain electrodes (|*V*_ds_|). In contrast, the mixing is minimized when |*V*_ds_|is larger than |*V*_gs_|. Furthermore, the larger the |*V*_gs_| potential is, the stronger the microvortices at the source-gate electrodes are due to the stronger field intensities. This explains why the operating conditions (Table 1) corresponding to Fig. 1c lead to the best mixing performance as semi-quantitatively and experimentally established in the future sections. The field-effect micromixer is insensitive to the voltage polarity and thus, only absolute potential differences are important. Additionally, the source and drain electrodes are essentially symmetric and can be interchanged. Hence, |*V*_gd_| potential difference shall be considered (instead of |*V*_gs_|) to predict micromixer behavior if the alternate electrode assignment is assumed.

## Materials and methods

### Microfluidic platform fabrication

The microfluidic platform consisted of gold electrodes on the device floor and a SU-8 fluidic channel with a thickness of 30 μm, a width of 160 μm, and a length of 8 mm. The device fabrication involved patterning gold electrodes on a Borosilicate glass slide (SCHOTT North America, Inc., Elmsford, NY). The glass slide was cleaned using acetone, isopropyl alcohol (IPA), and DI water. Next, photoresist AZ5214E was spin coated to form a 1.5 μm thick layer followed by soft baking at 110 °C. Then, the sample was undergone UV exposure with a dose of 20 mJ cm^−2^ and reversal bake at 110 °C for 2.5 min. Flood exposure was followed at 450 mJ cm^−2^, and developing was done in MIF720 developer for 30 s. Following the photolithography, 20 nm of titanium and 80 nm of gold were deposited using e-beam evaporation. The electrode fabrication was completed by sonication in Microposit Remover 1165 at 70 °C for 30 min.

Next, the glass substrate was cleaned with acetone and IPA, and rinsed with DI water followed by one-hour dehydration bake at 150 °C in a vacuum oven. The fluidic channel was formed by patterning a 30 μm thick SU-8 2015 layer. First, SU-8 2015 was spin coated, and soft baked at 67 °C and 97 °C for 5 and 10 min, respectively. The substrate was then exposed at 100 mJ cm^−2^ and baked at 67 °C for 2 min and 97 °C for 5 min. The sample was developed for 75 s in the SU-8 developer, and hard baked at 150 °C for 10 min to relieve surface cracks and to further harden the SU-8 film.

Finally, the flow channel was sealed by bonding a flat PDMS piece with inlet/outlet vias to the SU-8 layer on the substrate. First, (3-Aminopropyl) triethoxysilane (APTES) (Sigma Aldrich, St. Louis, MO) was vacuum deposited onto the substrate with the SU-8 layer for 1 h. The PDMS layer was plasma treated to activate the surface with oxygen groups immediately before bonding with the substrate. The bonding completed by heating the assembly on a hotplate at 100 °C for 8 h under 20N force.

### Experimental setup

The mixing experiments were performed by infusing DI water and Fluorescein dye solution using a standard infuse/withdraw syringe pump (11 Elite Programmable Syringe Pump, Harvardapparatus, Inc.). The Fluorescein solution was prepared by dissolving 2 mg of Fluorescein powder (Sigma-Aldrich, Inc.) in 1 ml of Acetone and supplementing with 300 ml of DI water followed by filtration using a 0.4 μm membrane syringe filter (Corning, Inc.). The Fluorescein solution had a conductivity of 20 µS cm^−1^ measured with a conductivity meter (HI98303, Hanna Instruments, Woonsocket, RI). The image acquisition setup consisted of an inverted microscope (TE-2000E, Nikon) equipped with a CCD camera (Retiga-2000R, Nikon) and Nikon NIS-Elements D software. The voltages were supplied by a 2-input/2-output function generator (AFG3200C, Tektronix, Inc.) at load impedance setting.

The lipid solutions for making liposomes as a proposed application for the micromixer were prepared by dissolving 2 mg of DPPC (1,2-dipalmitoyl-sn-glycero-3-phosphocholine) and 0.5 mg of Cholesterol in 10 mL of reagent alcohol (90% ethanol, 5% methyl alcohol, and 5% IPA) giving a final concentration of 0.2 mg/mL of DPPC with a 1:0.25 ratio of DPPC : Cholesterol. This ratio remained constant throughout all experiments. All lipid formulations were stored in glass vials (VWR International Radnor, PA, USA) with aluminum covered caps and stored at 4 °C until use. The lipid solutions and DI water were brought to room temperature before use in the microfluidic device.

### Mixing characterization

The mixing performance was characterized by measuring pixel intensities across the width of the channel (150 µm from where electrodes end) and calculating the mixing index (MI) where 1 and 0 represent perfectly mixed and unmixed states, respectively. The MI was computed by taking the ratio of the standard deviation of pixel intensities, *σ*, to the average of pixel intensities in the mixed state, $\bar I$, according to the formula below^50^:

3 $${\mathrm{MI}} = 1 - \frac{\sigma }{{\bar I}} = 1 - \frac{{\sqrt {\frac{1}{N}\mathop {\sum }\nolimits_{i = 1}^N (I_i - \bar I)^2} }}{{\bar I}}$$

where *I*_*i*_ represents the local pixel intensity and *N* is the total number of pixels.

## Results and discussion

### Mixing performance: frequency and voltage effect

As indicated in Eq. 1, the ACEO flow velocity is a strong function of frequency and voltage amplitude^35^. To find the optimal frequency for mixing, the mixing indices for the frequency range of 600 Hz–200 kHz were considered for each biasing scheme. Figure 2a presents the frequency response of the micromixer when two streams of DI water and Fluorescein solution were infused at a confluent flow rate of 4 µL/min with an excitation voltage of 10 V_pp_. At low frequencies (<1 kHz), unsteady and unpredictable flow patterns were observed. Moreover, prolonged operation in the order of minutes generated gas bubbles due to electrolysis and Faradaic reactions. At larger frequencies (>1 kHz), the mixing of streams was stable and peaked at 40–60 kHz frequency range. The mixing dependence on voltage was also evaluated by biasing the micromixer at the optimal frequency (40 kHz) and stepping up voltage from 2 to 10 V_pp_ in 1 V steps (Fig. 2b). The mixing degree increased as the voltage level increased which is in agreement with the theoretical prediction (Eq. 1) and previous literature^35^. An operating voltage of 10 V_pp_ (limited by the function generator) was selected for the rest of the experiments.

At the peak voltage of 10 V_pp_, bubbles were observed at frequencies below 1 kHz reaching the DC limit. These bubbles were large enough to clog the channel and severely disturb the flow streams, which required a high-pressure flow to remove them from the mixing channel. The source of these bubbles is the Faradaic reactions that generate ions periodically in time at the electrodes following Faraday’s law. Although the presence of bubbles is a strong indicator of Faradaic reactions taking place on the electrodes, Faradaic reactions do not necessarily result in gas formation. Reversible electrode dissolution and deposition often do not cause gas bubbles^51^. Moreover, it is possible that the gas molecules generated in each half-cycle are insignificant such that they get dissolved without nucleating macroscopic bubbles. Negligible pH gradients as a result of Faradaic process may also not materialize as the reaction products can be consumed in the reverse reaction within the next half-cycle of an AC signal. With this understanding of the Faradaic process, it is evident that macroscopic bubbles and intensely unstable flows observed at frequencies below 1 kHz were triggered by strong Faradaic reactions. To mitigate these problems, AC signals at frequencies much higher than the inverse Faradaic reaction time should be applied to induce capacitive charging on electrodes for realizing ACEO flow, the theory of which was described earlier^52^. However, since it is possible to have Faradaic reactions without discernable physical manifestations, further understanding of flow mechanism under each phenomenon (Faradaic charging vs capacitive charging) is required.

The time-periodic Faradaic charging is qualitatively different from capacitive charging. In Faradaic charging, coions are produced at the electrodes by electrochemical reactions instead of counterions moving from the bulk to the surface of electrodes by electromigration. As a result, in a Faradic reaction, coions which have the same polarity as the electrodes amplify the external field instead of screening it. Due to this effect, it is expected that Faradaic charging produces flow velocities that increase monotonically and approach a constant asymptote at low frequency with the assumption that the reaction is reversible and reaches equilibrium within a half-cycle^51^. This behavior is different from ACEO flow based on capacitive charging, where the fluid velocity peaks at a characteristic frequency. In other words, the frequency dependence of the velocity has a bell-shaped profile in capacitive charging^35^. As can be observed in Fig. 2a, the frequency dependence of the MI had a bell-shaped curve with a characteristic frequency at which optimized mixing was achieved, an indication that ACEO streams were at their peak velocity. Based on this analysis, it is reasonably safe to assume Faradaic reactions were not significant in this study.

Aside from Faradaic charging, Joule heating and the subsequent ACET flow may also be present in certain experimental conditions. The Joule heating produces a temperature (*T*) field that depends on liquid conductivity and the voltage amplitude through Δ*T* *~* *σV*^2^*/2k*, with *k* being the fluid heat conductivity^53^. The increase in temperature produces temperature gradients, which lead to conductivity $\left( {\nabla \sigma = (\partial \sigma /\partial T)\nabla T} \right)$ and permittivity $\left( {\nabla \varepsilon = (\partial \varepsilon /\partial T)\nabla T} \right)$ gradients within fluid bulk. The conductivity and permittivity gradients lead to variations in net charge density generating an electrostatic body force. Studies by Green et al.^54^ and Castellano et al.^53^ suggest that, for voltages smaller than 10 V (20 V_pp_) and conductivities less than ~10^−2^ Sm^−1^ (10^−4^ Scm^−1^), Joule heating is insufficient for the generation of temperature gradients to account for observed flow velocities attributed to ACEO flow. Another theoretical and experimental investigation of ACET flow by Loire et al. also indicates that, at a conductivity of 40 × 10^−4^ Scm^−1^ and voltages <10 V_pp_, insignificant flow velocities less than 0.25 µm s^−1^ are generated^55^. Noting that the liquid conductivities in these studies are at least two orders of magnitude larger than the current study, it is reasonable to assume that observed flows are solely sourced by ACEO and not ACET phenomenon.

### Phase-controlled mixing

**Fig. 3:**
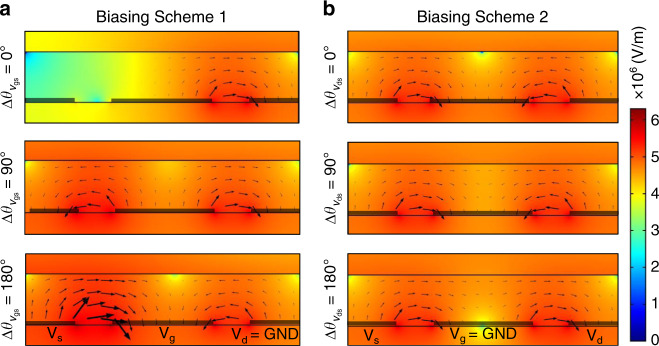
Electric field phasor analysis. **a** Logscale electric field intensity for biasing scheme 1 at different phase lag values on the source-gate electrodes. **b** Logscale electric field intensity for biasing scheme 2 at different phase lag values on the source-drain electrodes

In the proposed electrode setup, the electric field intensity and distribution were tunned by imposing a phase lag between electrodes, which directly impacted the ACEO flow velocities and the mixing degree. Thus, the electric field distribution was studied by simulating a 2D cross-section of electrodes including the glass substrate, gold electrodes, fluid (DI water), and the PDMS cover using COMSOL 5.2 (Burlington, MA). The simulations were performed by employing the Electric Current module in the frequency domain for which AC signals (10 V_pp_ at 40 kHz) were applied according to the biasing schemes introduced earlier (Fig. 1b–e). The logscale electric field intensities were evaluated for better visualization of electric field variations across electrodes. For each biasing scheme, the phase shifts of 0°, 90°, and 180° were assumed. Figure 3a shows phasor analysis of electric field intensity with current density streamlines corresponding to the biasing scheme 1. The electric field simulations qualitatively elucidate the mixing dependence on the phase lag between the driving voltages. As shown in this figure, when the gate and source electrodes are in-phase $\left( {\Delta \theta _{V_{\text{gs}}} = 0^\circ } \right)$, the electric field intensity is very weak at the source-gate electrode gap. This should lead to small fluid velocities at this gap, which effectively put the micromixer in an off state. However, as the phase lag between the source-gate electrodes is increased, the field intensity is enhanced that is expected to induce stronger flow fields. This is evident in Fig. 3a when a phase lag of 90° $\left( {\Delta \theta _{V_{\text{gs}}} = 90^\circ } \right)$ is imposed on the source-gate electrodes. Finally, when the gate and source electrodes are completely out of phase $\left( {\Delta \theta _{V_{\text{gs}}} = 180^\circ } \right)$, the electric field intensity is maximized for the given potentials and hence, maximized mixing performance is expected.

Similarly, the micromixer behavior for the biasing scheme 2 can be explained by analyzing the field intensities at different phase lags between the source and drain electrodes. The electric field phasor analysis in Fig. 3b indicates that electric field intensity peaks for in-phase voltages $\left( {\Delta \theta _{V_{\text{ds}}} = 0^\circ } \right)$. At a phase lag of 90°, the field intensity slightly decreases especially on the center electrode. For the antiphase voltages $\left( {\Delta \theta _{V_{\text{ds}}} = 180^\circ } \right)$, however, the reduction in electric field strength on the center electrode is more pronounced while the field intensities at the source-gate and drain-gate electrodes are relatively unchanged. Accordingly, it is expected to observe optimal mixing with in-phase signals and minimal mixing with antiphase voltages.

**Fig. 4:**
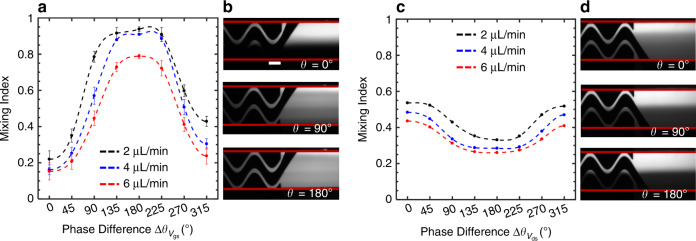
Phase-controlled mixing. **a** MI versus phase difference for the biasing scheme 1 with $\Delta \theta _{V_{\text{gs}}}$ changing. **b** Grayscale fluorescent images of the channel output showing the mixing of DI water (dark) and fluorescein solution (bright) corresponding to plot in (**a)** for the confluent flow rate of 4 µL/min. **c** MI versus phase difference for the biasing scheme 2 with $\Delta \theta _{V_{\text{ds}}}$ altering. **d** Grayscale fluorescent images of DI water and fluorescein solution corresponding to plot in (**c**) for the confluent flow rate of 4 µL/min

The findings from the electric field simulations and the mixing dependency on the imposed phase lag were experimentally validated for various flow rates. Figure 4 shows the mixing indices versus phase lag for the two discussed biasing schemes. For the biasing scheme 1, the mixing performance enhanced as the imposed phase lag on the driving electrodes was increased from 0° to 180° for all flow rates (Fig. 4a). The increase in mixing indices by increasing the phase lag is in agreement with the simulation analysis presented previously. Figure 4b illustrates the grayscale fluorescent images of the actual device for phase lags of 0, 90, 180 degrees corresponding to the confluent flow rate of 4 µL/min in Fig. 4a. At 0° phase lag, barely any mixing occurs between the two streams. At 90° phase lag, partial mixing takes place. When the voltages are antiphase, the two streams are completely mixed.

For the biasing scheme 2 (Fig. 4c), an opposite response to phase lag with less intensity was observed which agrees with the electric field simulation analysis. Figure 4d provides the grayscale fluorescent images of the micromixer representing the confluent flow rate of 4 µL/min in Fig. 4c. At 0° phase lag, the best mixing performance was achieved. The mixing slightly decreased for 90° phase lag and reached the lowest level at 180° phase lag.

Finally, the performance of the proposed micromixer was compared with the existing electrokinetic micromixers based on ACEO induced flow actuation. Since different studies vary widely in the design of microfluidic channels, the maximum volumetric flow rates sustaining MIs of 0.8 or higher as reported in the literature were converted to average linear velocities for a meaningful comparative analysis. Accordingly, micromixers that can operate at larger velocities to attain a threshold mixing performance are better mixers. Table 2 lists various ACEO micromixers with the pertinent reported channel and voltage parameters. As shown in this table, the proposed micromixer (a) achieves the highest linear velocity compared with other micromixers. Moreover, the applied voltage is the same as or smaller than other micromixers except that of micromixer in (d). Thus, a true comparison between the current study and the micromixer in (d) is not feasible. However, it is notable that the micromixer in (d) operates at the low frequency of 1 Hz, which makes it very likely to cause Faradaic reactions at marginally higher liquid conductivities or prolonged voltage excitation.

**Table 2:** Comparison of the proposed micromixer with the existing electrokinetic micromixers based on ACEO fluid actuation

Micromixer	Channel width (µm)	Channel height (µm)	AC voltage parameters	Volumetric flow rate (µL/min)	Average linear velocity (mm/s)
a	160	30	10 V_pp_ at 40 kHz	4	13.9
b^25^	120	40	20 V_pp_ at 1 kHz	2	6.9
c^23^	100	44	20 V_pp_ (−2.5 DC) at 100 kHz	2	7.6
d^22^	140	60	5 V_pp_ at 1 Hz	1.25	2.5
e^24^	400	100	20 V_pp_ at 2 kHz	2	0.83
f^18^	180	100	10 V_pp_ at 500 Hz	NA	2

### Application of phase-controlled micromixing

To assess the applicability of the proposed micromixer in chemical synthesis applications, fabrication of nanoscale liposomes was carried out. Liposomes are drug delivery agents that consist of a lipid bilayer shell and an aqueous core that can encapsulate various drugs and nutrients^56^. Microfluidic-based liposome synthesis is achieved through the process of nanoprecipitation (or solvent displacement) when coflowing streams of aqueous phase mix with the reagent-containing solvent^57^. The liposomes are formed by hydrophobic forces through the process of self-assembly of lipid precursors into vesicles. The most widely utilized and studied microfluidic platforms for liposome synthesis involve hydrodynamic flow focusing (HFF) wherein a center stream containing the precursors is narrowed by sheath streams to enhance diffusion-based mixing at the liquid boundaries^58^. However, effective mixing at the boundaries necessitates very high flow rate ratios (FRR) of the sheath streams to the sample stream (up to 50), which limit the product throughput and yield. Thus, recent studies have investigated nanoparticle synthesis employing other types of passive or active mixers to achieve improved throughput and homogeneous batches by enhancing mixing. In-droplet mixing^59^ and acoustic streaming^60^ are two examples of such mixers. While both techniques are effective methods for the on-chip nanoparticle synthesis, they suffer from drawbacks that limit their application. The droplet-based techniques (mostly oil-based) demand the use of surfactants for droplet stabilizations that may contaminate the products. The separation of water and oil phases further adds to the post-processing steps. On the other hand, acoustic-based methods require the use of elastomeric microchannels for the vibration of protruding fingers to introduce acoustic microstreaming, which demand the application of high voltages (tens of volts) for decent throughputs. In that context, electrical-based methods can provide a viable approach for nanoparticle synthesis that operate with low voltages without any moving parts or the addition of extra chemicals. Most importantly, the nanoprecipitation technique generally employs DI water and low conductivity solvents (e.g., ethanol, isoproponal, acetonitrile, etc.) that are ideal for electrical-induced fluid actuation mechanisms since the likeliness of Faradaic reactions and product contamination is very slim.

**Fig. 5:**
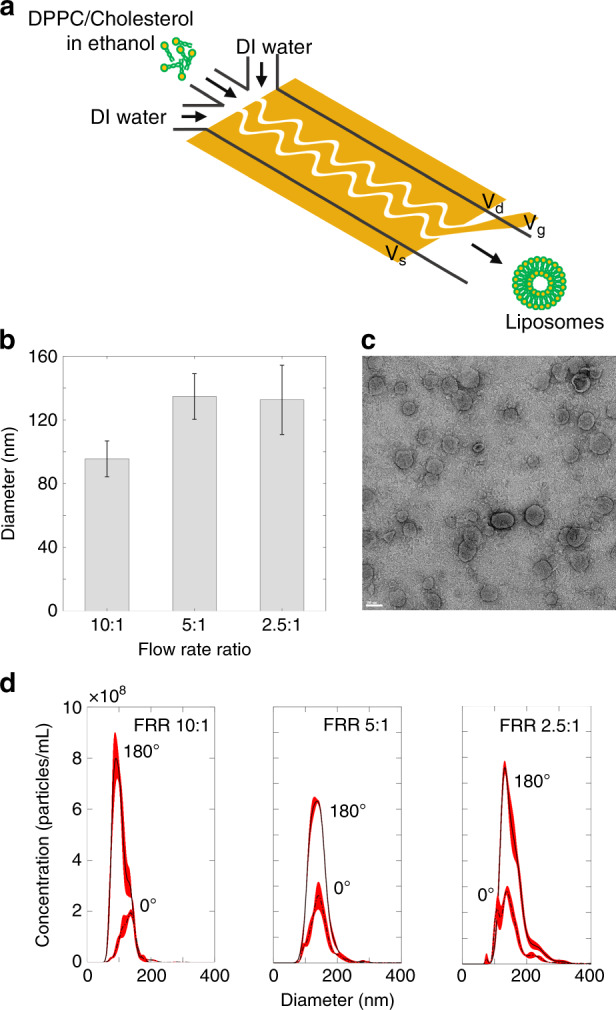
Nanoscale liposome synthesis. **a** Schematic illustration of the modified device for nanoparticle synthesis. **b** Liposome average diameter versus different FRR. The error bars indicate the average standard deviations of size distributions in three NTA runs of a single batch, **c** A TEM image of synthesized liposomes (FRR 5:1). The scale bar is 100 nm. **d** Size distribution of synthesized nanoparticles at different FRRs for phase shifts of $\Delta \theta _{V_{\text{gs}}} = 180^{\circ}$ and $\Delta \theta _{V_{\text{gs}}} = 0^\circ$. (*n* = 3, total flow rate: 200 µL/min)

Accordingly, the proposed micromixer was modified to accommodate three microfluidic inlets to introduce two streams of an aqueous phase (DI water) and a stream of a solvent (ethanol) containing lipid precursors (Fig. 5a). Liposomes were formed through the process of self-assembly of hydrophobic lipid tails when streams of lipid-containing ethanol and DI water mixed. For such biphasic liquid systems, the mixing mechanism is no longer based on ACEO, instead, electrohydrodynamic instabilities facilitate the mixing of ethanol and DI water streams. This is due to the existence of a sharp discontinuity in electrical parameters of DI water and ethanol in the presence of an electric field that accumulates monopolar charges at their interface. The electrical shear forces acting on the charged interface then give rise to fluid motion^38^. Since the flow actuation mechanism is different from ACEO, the micromixer frequency response at the peak voltage of 10 V_pp_ was characterized by considering different FRR of sheath streams to sample stream of 10:1, 5:1, and 2.5:1 (Fig. S4a). Although the electrohydrodynamic mixing mechanism is distinct from that of ACEO flow, the principle of phase-controlled tunning of electric field intensity and mixing degree is still valid.

The liposomal batches were synthesized by operating the micromixer at optimal biasing conditions (scheme 1) and the frequency (1 MHz) at three different FRRs of water to lipid-containing solvent. The average size and size distribution of batches were measured using a Nanoparticle Tracking Analysis (NTA) equipment. Figure 5b shows the liposome diameter versus different FRRs, and Fig. 5c is a TEM image of synthesized liposomes at a FRR of 5:1. The effect of mixing enhancement on the size characteristic of liposomal batches was assessed by operating the micromixer at the optimal mixing condition with a phase shift of 180° and the minimal mixing condition with a phase shift of 0°. Figure 5d shows size distributions of batches produced at different FRRs considering phase lags ($\Delta \theta _{V_{\rm{gs}}}$) of 180° and 0° for each FRR. As observed in this figure, the monodispersity and concentration of batches were significantly improved when the micromixer was run with optimal mixing at a 180° phase shift.

## Conclusion

In this article, a novel phase-controlled electrokinetic micromixer based on ACEO-generated microflows was introduced, and its working mechanism was elaborated and analyzed based on electric field simulations and experimental characterizations. By modulating the phase lag between the driving voltages at constant amplitudes, the electric field distribution and intensity can be altered leading to different AC electroosmotic slip velocities and mixing performances. It is essential to note that the phase relation that leads to the best performance is a strong function of electric field distribution, which can be specified by the electrode orientation and biasing layouts. The utility of the proposed micromixer was extended to the mixing of reagents in the process of nanoprecipitation to produce lipid nano-vesicles. We only presented the preliminary results for nanoparticle synthesis, and those early results have shown promising outcomes to benefit from electrical-assisted techniques for nanoparticle formation. Further detailed studies are being carried out to fully characterize the mixing mechanism for water-solvent-water coflowing streams^61^ and nanoparticle synthesis^61,62^ applying the electrohydrodynamic-driven micromixing.

## Supplementary information

Phase-controlled Mixing - Biasing 1

Phase-controlled Mixing - Biasing 2

Supporting Information
