# Supplementary material for: Phase-controlled field-effect micromixing using AC electroosmosis
Source: Microsyst Nanoeng. 2020 Jul 27;6:60. doi: 10.1038/s41378-020-0166-y (PMC8433414; doi:10.1038/s41378-020-0166-y)
Supplement: Supplementary file 3 — Supporting Information [file 41378_2020_166_MOESM3_ESM.pdf]

Phase-Controlled Field-Effect Micromixing Using AC Electroosmosis

Paresa Modarres and Maryam Tabrizian*

Biomedical Engineering Department, Faculty of Medicine, McGill University, 3775 University Street, Montreal, Quebec, Canada H3A 0G4

Supporting Information

*Corresponding author at:

Department of Biomedical Engineering, Faculty of Medicine, McGill University, 3775 University Street, Montreal, Quebec H3A 0G4, Canada.
Tel: 514-398-8129

Email addresses:
paresa.modarres@mail.mcgill.ca (Paresa Modarres)
maryam.tabrizian@mcgill.ca (Maryam Tabrizian)

Device geometry

The proposed micromixer consists of a Y-shaped microchannel with three-finger electrodes that are shaped sinusoidally (s-shape) running parallel to the main channel (Fig. S1a). To compare the efficacy of the proposed electrode geometry to that of rectangular-shaped (r-shape) electrodes, a micromixer with straight electrodes (Fig. S1b) was also fabricated and evaluated. For both designs, L, W, and H correspond to the electrode length, channel width, and channel height, respectively. The gate, source, and drain electrodes widths are designated as w_g, w_s, and w_d, respectively. The spacing between each electrode pair is d. The design parameters for the s-shape and r-shape micromixers are listed in Table S1.

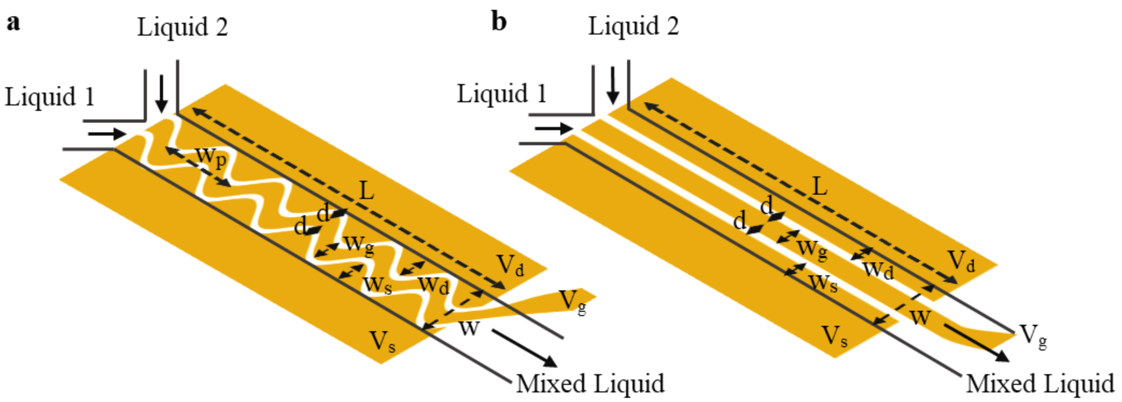

Figure S1. **a** S-shape **b** and r-shape electrode micromixer design parameters.

Table S1. Geometrical parameters for the s-shape and r-shape electrodes and the microfluidic channel.

**Parameter**	**Value (μm) S-shape**	**Value (μm) R-shape**	**Description**
w_g	60	60	Gate electrode width
w_d	0 – 60 (variable)	30	Drain electrode width
w_s	0 – 60 (variable)	30	Source electrode width
w_p	160	-	Electrode periodicity
d	20	20	Electrode spacing
w	160	160	Channel width
h	30	30	Channel height
L	3000	3000	Electrode length

Mixing performance: device geometry

Unlike the majority of prior electrokinetic-based micromixers that encompassed rectangular electrode patterns symmetric to the flow direction, the altering configuration of the sinusoidally shaped electrodes introduces asymmetric vortices with respect to the interface of the incoming fluid streams along the mixing length. To assess the mixing enhancement attained with the sinusoidal electrode geometry, a micromixer with parallel rectangular electrodes was fabricated and characterized (Fig. S1b). All parameters including electrode length, channel width, channel height, electrode spacing, and gate electrode width remained constant for both designs for a proper comparison.

Fig. S2a shows the mixing indices versus frequency for a confluent flow rate of 4 μL/min and excitation voltages of 10 V_{pp} using biasing scheme 1. As it is observed in this figure, the maximum mixing for the r-shape electrode geometry was obtained at a frequency range of 10-20 kHz. Thus, for the r-shape electrode, the mixing variation with voltage was characterized at 10 kHz and is shown in Fig. S2b. Overall, it can be concluded that in r-shape electrode geometry the mixing was significantly reduced corresponding to an average of 187% decrease in the peak mixing index with the biasing scheme 1.

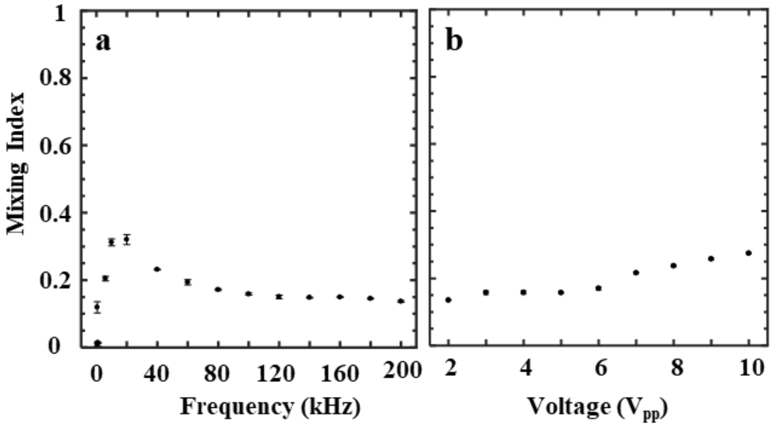

Figure S2. **a** Mixing index versus frequency for r-shape electrode geometry (V = 10 V_{pp}, Biasing scheme 1). **b** Mixing index versus voltage for r-shape electrodes. All experiments were performed with a total flow rate of 4 μL/min.

Nanoparticle synthesis

The device geometry was modified to allow the injection of two aqueous streams and a precursor-containing solvent to demonstrate the applicability of the proposed micromixing mechanism and platform for nanoparticle synthesis. The modified geometry consists of the same s-shape electrode pattern with a widened microfluidic channel with three inlets. The schematic illustration of the modified design is shown in Fig. S3 and the relevant geometrical parameters are listed in Table S2.

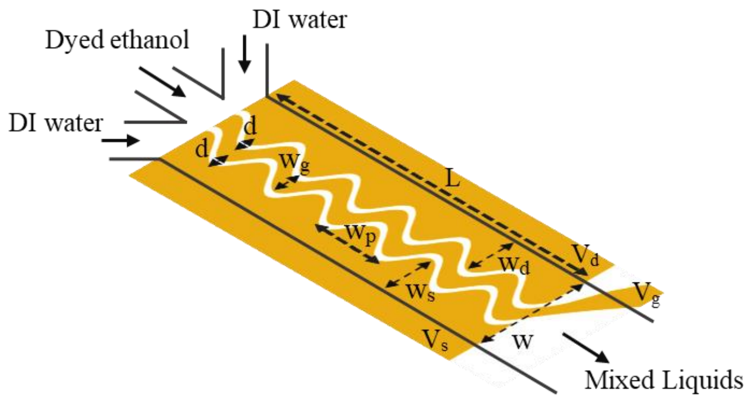

Figure S3: The modified platform for nanoparticle synthesis

The frequency response of the micromixer for a water/ethanol/water flow system was assessed by injecting Rhodamine-B dyed ethanol solution and DI water. The excitation voltages remained constant at 10 V_{pp} and the frequency was varied ranging from 1 kHz to 20 MHz. Furthermore, the mixing quality for three different flow rate ratios (FRR) of water to ethanol stream was examined. The frequency response and the effect of FRR on the mixing performance are presented in Fig. S4a. According to mixing indices in Fig. S4a, frequencies above 100 kHz for all FRRs resulted in optimized mixing. Next, the effect of the total flow rate (TFR) on the mixing

index was evaluated by operating the mixer at the optimized frequency of 1 MHz and a voltage of 10 V_{pp}. Fig. S4b shows the mixing performance corresponding to TFR values ranging from 10 µL/min to 400 µL/min at different FRRs.

Table S2: Geometrical parameters of the electrodes and the microfluidic channel for the modified platform.

**Parameter**	**Value (µm)**	**Description**
w_g	60	Gate electrode width
w_d	0 – 120 (variable)	Drain electrode width
w_s	0 – 120 (variable)	Source electrode width
w_p	160	Electrode periodicity
d	20	Electrode spacing
w	280	Channel width
h	60	Channel height
L	3000	Electrode length

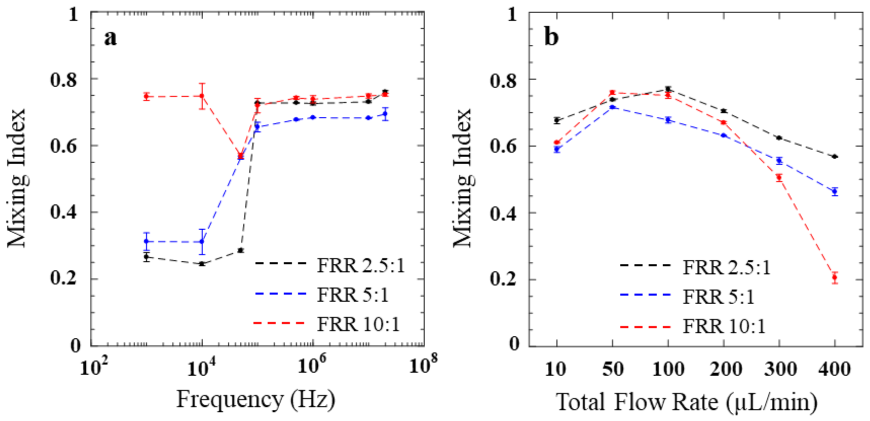

Figure S4. **a** Mixing index versus frequency at different FRRs of DI water to ethanol. (V: 10 V_{pp}, TFR: 50 µL/min) **b** Mixing index versus total flow rate at different FRRs of DI water to ethanol. (V: 10 V_{pp} at 1 MHz)
